# Effects of Sociodemographic Variables and Depressive Symptoms on MoCA Test Performance in Native Germans and Turkish Migrants in Germany

**DOI:** 10.3390/ijerph18126335

**Published:** 2021-06-11

**Authors:** Görkem Anapa, Mandy Roheger, Ümran Sema Seven, Hannah Liebermann-Jordanidis, Oezguer A. Onur, Josef Kessler, Elke Kalbe

**Affiliations:** 1 Neuropsychology and Gender Studies & Center for Neuropsychological Diagnostics and Intervention (CeNDI), Department of Medical Psychology, Faculty of Medicine and University Hospital of Cologne, University of Cologne, 50937 Cologne, Germany; goerkem.anapa@gmail.com (G.A.); uemran.seven@uk-koeln.de (Ü.S.S.); hannah.liebermann-jordanidis1@uk-koeln.de (H.L.-J.); 2 Department of Neurology, University Medicine Greifswald, 17489 Greifswald, Germany; mandy.roheger@med.uni-greifswald.de; 3 Department of Neurology, Faculty of Medicine and University Hospital of Cologne, University of Cologne, 50937 Cologne, Germany; oezguer.onur@uk-koeln.de (O.A.O.); josef.kessler@uk-koeln.de (J.K.)

**Keywords:** Montreal Cognitive Assessment (MoCA), cognition, Turkish migrants, predictors

## Abstract

The validity of the Montreal Cognitive Assessment (MoCA) in migrants is questionable, as sociodemographic factors and the migration process may influence performance. Our aim was to evaluate possible predictors (age, education, sex, depression, and migration) of MoCA results in Turkish migrants and Germans living in Germany. Linear regression models were conducted with a German (*n* = 419), a Turkish (*n* = 133), and an overall sample. All predictor analyses reached statistical significance. For the German sample, age, sex, education, and depression were significant predictors, whereas education was the only predictor for Turkish migrants. For the overall sample, having no migration background and higher education were significant predictors. Migration background and education had an impact on MoCA performance in a sample of German and Turkish individuals living in Germany. Thus, culture-specific normative data for the MoCA are needed, and the development of culture-sensitive cognitive screening tools is encouraged.

## 1. Introduction

Migration is an increasing phenomenon worldwide [1], and in Germany, Turkish migrants represent the largest population group with a migration background [2]. There is an increasing number of older people being diagnosed with dementia in general [3,4], and a high number of people with migration background living in Germany with dementia [5]. Therefore, valid screening of cognitive impairment and dementia in this population group is important and will become even more relevant with regard to the demographic change [6,7]. Migration is a critical life event and causes changes concerning different life areas (e.g., socioeconomic status, social chances and risks, and health chances and risks), which may have an influence on health and cognition [8,9]. For several countries, studies have shown that general health status and cognitive test performance can be lower in individuals with a migration background than in those without [8,9]. For example, in Germany, language barriers and low educational level of immigrated persons are known to negatively influence access to the German health care system [10]. Moreover, recent studies show correlations also between level of acculturation and health-related quality of life [11,12]. However, cognitive screening tools that provide normative values for migrants are lacking.

One of the most frequently used cognitive screening tools in both Germany and worldwide is the Montreal Cognitive Assessment (MoCA; [13]). Yet, limitations exist concerning the assessment and results of the MoCA in general and for migrants in particular, which affect its interpretation and decrease its diagnostic accuracy. For example, test scores are corrected for education, but not for age and sex, although all of these variables have been demonstrated to have an impact on test performance [14,15,16,17]. Moreover, depressive symptoms may influence cognitive performance and, in fact, have been shown to decrease MoCA performance [18,19]. Concerning Turkish people living in Germany (and migrants more generally), it is possible that factors associated with the process of migrating also influence MoCA outcomes. Notably, migration is known to be a stressful event with manifold challenges, and previous research indicates that stress is a possible etiological factor for cognitive impairment and Alzheimer’s disease [20,21,22].

In terms of language barriers, a Turkish version of the MoCA already exists. However, it is questionable whether it is an appropriate and culture-sensitive tool to assess the cognitive status of Turkish migrants living in Germany, as it was validated in a Turkish population living in Turkey and did not consider the possible bias of specific characteristics of this population, including the migration processes and its consequences [23].

Taken together, there is a need to investigate possible influencing factors on MoCA test performance in individuals with a migration background, particularly sociodemographic variables (e.g., age, sex, and education), depression, and the migration process. To address this issue, we investigated MoCA test performance in native Germans and Turkish migrants aged 50 years or older living in Germany by testing them in their mother tongue (i.e., with the German and Turkish version of the screening tool). Next, the sociodemographic variables of age, education, and sex, depressive symptoms, and migration background were analysed as potential predictors of performance. Our hypotheses were that (1) age, sex, education, and depression are predictors for cognition measured by the MoCA in both the German and the Turkish samples. Furthermore, we expect that “country of origin” is also a significant predictor for MoCA performance (2).

## 2. Materials and Methods

The present cross-sectional study was conducted with healthy native German (*n* = 419) and Turkish people living in North Rhine–Westphalia, Germany (*n* = 133) as part of the TRAKULA project, which aimed to develop a culture-sensitive nonverbal test battery. Recruitment took place in 2016 and 2017 after the study was approved by the ethical committee of the University Hospital Cologne (16-249). The sample can be described as old-aged according to the World Health Organization (WHO), since the majority of the subjects were over 55 years of age [24]. Participants were recruited at different institutions in North Rhine–Westphalia. During recruitment, attention was paid to a balanced and diverse selection of contact points for people of Turkish origin. Individuals were recruited mainly in Cologne and the surrounding area, where many people with a Turkish migration background live, for example, in cultural institutions, mosques, or places where the Turkish community is commonly found. Furthermore, advertisements were placed in doctors’ offices and on our clinic website. Written informed consent was obtained from each participant. All participants completed questionnaires assessing sociodemographic details. Testing and recruiting were conducted in the participants’ mother tongue by German–Turkish bilingual and bicultural psychologists (authors G.A. and Ü.S.S.) and trained bilingual students of medicine or psychology supervised by authors G.A. and Ü.S.S.

### 2.1. Inclusion and Exclusion Criteria

For all participants, inclusion criteria were a residency in Germany, being native German or being native Turkish and having a migration background, aged 50 years or older, normal, only slightly restricted, or corrected-to-normal vision and hearing, and the provision of written consent to participate in the study. Exclusion criteria were self-reported cognitive impairment or other past or current diagnosed neurological or psychiatric illnesses and cognitive disorders.

### 2.2. Assessment of Cognition and Depression

The MoCA [13] was administered using either the German or Turkish version [23,25]. Both versions have a maximum of 30 points and differ only in two subtests: memory (different words) and word fluency (letter “K” instead of “F”). The cultural adjustment that was made concerns the word “church” in the memory subtest, which was replaced by the word “mosque”. The awarding of points in the individual subtests corresponds to the original version of the MoCA. Participants are classified as cognitively impaired when reaching less than 26 points in the German [25] and less than 21 points in the Turkish version [23]. The German version provides an education adjustment of one point for people who received less than 12 years of school education, whereas the Turkish version does not use an education adjustment, as proposed by Selekler et al. (2010) [23]. The cognitive domains tested in the MoCA are visuospatial/executive functions, naming, verbal short-term memory, attention, language, abstraction, and verbal long-term memory [13].

Furthermore, the participants received the German or Turkish version of the Geriatric Depression Scale Short Form (GDS-15; [26]) to assess depressive symptoms. The instrument consists of 15 yes/no items. Each answer counts as one point, and a maximum score of more than 5 points indicates possible, 6 to 10 points moderate, and 11 to 15 points severe depressive symptoms [26].

### 2.3. Statistics

Statistical analyses were performed using IBM SPSS Statistics 25 for Windows (IBM Corp, Armonk, NY, USA). Normal distribution was tested using the Kolmogorov–Smirnov test. Possible differences in demographic data (age, sex, and education) and depressive symptoms between the two groups (German and Turkish participants) and within groups were analysed using t-tests, or chi-square tests, where appropriate, each with a significance level of α = 0.05. G*Power (http://www.gpower.hhu.de, accessed on June 2018) was used to estimate the achieved power with a post hoc analysis [27].

To analyse predictors of MoCA performance, linear regressions were conducted (one for the German and one for the Turkish sample), with the total MoCA score as a dependent variable and simultaneous entry of the predictors of age, sex, education, and depression.

To further analyse migration as a possible predictor of MoCA performance, an additional regression analysis was calculated for the combined German and Turkish sample, including age, sex, education, depression, and migration as possible predictors. Due to different sample sizes, the German and Turkish samples were matched. For this purpose, the first 133 German participants (mean age: 60.71, SD: 8.71; mean years of education: 11.99, SD: 1.90) were included in the total sample size and ordered according to sex to ensure a uniform distribution of women and men participants in the overall regression analysis. The education correction was not included in total MoCA scores used in the regression. The assumptions of multiple regression models were checked according to the suggestions of Field [28].

## 3. Results

### 3.1. Sample Characteristics

Demographic characteristics and results of the MoCA and GDS are shown in Table 1. Participants in the Turkish sample were significantly younger, less educated, showed significantly lower scores in the MoCA, and were significantly more depressed than participants in the German sample. German women were significantly less educated and more depressed than German men, whereas Turkish women were significantly younger, less educated, and showed lower performance in the MoCA than Turkish men did.

### 3.2. Predictor Analysis

The regression model for the German sample is presented in Table 2. The entire regression model reached statistical significance (*F*(4, 412) = 25.77; *p* < 0.001). The explained variance of the model was 19.2% (R^2^ = 0.19; *p* < 0.001). Predictors for MoCA performance were more years of education (B = 0.36), younger age (B = −0.05), being male (B = −0.46), and lower levels of depression scores (B = −0.21).

Table 3 presents the regression model for the Turkish sample. This regression model was also significant (*F*(4, 128) = 6.25; *p* < 0.001), but in contrast with the German sample, the only significant predictor in the Turkish sample was more years of education (B = 0.33). The explained variance of this model was 13.7% (R^2^ = 0.137; *p* < 0.001).

The regression model for the overall sample of German and Turkish participants, which included the predictors age, sex, education, and depression as well as migration, was significant (*F*(5, 259) = 15.16; *p* < 0.001), with having no migration background (B = 1.14) and higher education (B = 0.34) as significant predictors. The explained variance of this model was 21.1% (R^2^ = 0.211; *p* < 0.001) (Table 4).

## 4. Discussion

In this cross-sectional study with healthy older German adults and healthy older adults with Turkish migration background living in Germany, we investigated the impact of the sociodemographic variables age, sex, education, and migration background as well as depressive symptoms on cognitive performance, operationalized with the broadly used MoCA screening test. The main results are that MoCA performance (i) in the German sample was predicted by age, sex, and education, (ii) in the Turkish sample was only predicted by education, and (iii) in an overall sample was predicted by education and migration background.

First, results indicate that the German and Turkish samples differ significantly in sample characteristics; however, this finding reflects the current state of research: regarding sociodemographic variables, Turkish people are younger and less educated, which is in line with the results of a representative statistical survey on individuals with migration background in Germany [2]. Furthermore, they are more depressed, which is also in line with a previous representative study [29,30]. Finally, MoCA total scores were lower, which was expected according to other results on the cognitive state of migrants in different countries [31].

Our finding that MoCA performance in the German sample was predicted by more years of education, younger age, being male, and less depressive symptoms is in line with recent investigations in other population-based cohort studies that found significant predictors of cognition were also age, sex, and education [32,33,34]. The fact that younger age predicts MoCA performance has been described before [35] and can be well explained by the normal process of aging, which is accompanied by structural and functional brain changes leading to age-associated cognitive decline [36]. Further, the impact of education on cognition per se and the MoCA is also well known [37]. In contrast, sex as a possible predictor has less consistently been demonstrated and needs more consideration in future research [32,33,38,39,40,41]. Notably, as a typical pattern, German women were less educated than men in our sample, which can partly explain the results. Finally, our finding that lower depressive symptoms predict MoCA performance in the present study matches a whole body of evidence supporting the negative impact depressive symptoms have on cognition [42] and, more specifically, in elderly people [19]. In summary, in light of our data and other available data, the fact that the MoCA only provides a correction for education—at least for the German version—seems insufficient, and further versions that correct for the major influencing factors will improve precision of the screening process [34].

For the Turkish sample, the only significant predictor for MoCA performance was education, again demonstrating the consistency of this influence factor on cognition. In a study by Demir and Özcan (2015) [43], education was also an influencing factor on MoCA test performance in a Turkish population, but only in the language subtest. It is interesting to note that no differences could be detected in the other subtests. One reason for this could have been the small sample size of the study (*n* = 50). In another study on MoCA performance in Turkish migrants living in Germany [44], education and age were significant predictors. A possible reason for the lack of a significant age effect in our sample is the narrow age range, as 86.5% of the Turkish migrants were between 50 and 69 years and only 13.5% were 70 years or older (Table 1), whereas in the study by Krist et al. (2017) [44], individuals aged between 20 and 69 years were included. Sex was not a significant predictor in either study. Interestingly, depression, which was not assessed by Krist and colleagues, was not a significant predictor of MoCA performance in our study, although our Turkish sample was more depressed than our German sample. Thus, the impact of depression on cognitive performance in this population needs further investigation.

Our overall regression analysis, including both German and Turkish samples, again identifies education as having a substantial impact on MoCA performance. However, having or not having a migration background was an even stronger predictor of MoCA scores, showing that the migration process itself or life circumstances as a migrant predicts lower performance. Notably, Turkish migrants have worse working conditions, socioeconomic status, and health states in comparison with German people. However, it has been proposed that due to the harmful experience of migration, Turkish migrants are affected by faster aging processes, and this aspect was related to the fact that the prevalence of dementia is increasing more among this population in comparison with native Germans [7]. All of these aspects may explain why the migration background has such an influence on MoCA results even in healthy individuals. Importantly, our data indicate that normative data for the specific group of migrants will enhance the diagnostic accuracy of cognitive screenings. This aspect is also supported by further research. In a comparative study in the USA, different cut-off values for the MoCA were examined depending on ethnicity. In the detection of the optimal cut-off value in MCI, the authors found a point discrepancy of 1–2 points among non-Hispanic Whites, Hispanics, and non-Hispanic Blacks and a point discrepancy of 3 points in the detection of dementia [31]. Another systematic review that examined the cross-cultural application of MoCA also found a wide range of cut-offs, within and across different cultures [45].

Some limitations must be considered when interpreting the results of our study. First, information about health status (neurological, cognitive, and psychiatric disorders) was only assessed by self-reports. We cannot ensure that the study population did not include cognitively impaired participants, especially when considering MoCA test scores, as the MoCA cut-off score used for the Turkish sample was as low as 21 points, following the normative study by Selekler et al. (2010) [23]. When comparing our Turkish control group with the control group of Selekler et al. (2010) [23], there are differences of about two points. In the control group of Selekler et al. (2010), the average MoCA score was 23.50 (SD: 3.73), whereas our Turkish population averaged 25.11 points (SD: 3.40). Therefore, further studies should exclude cognitive impairment and dementia with a more elaborate clinical examination. In addition, an elaborated neuropsychological assessment is needed to replicate our findings to ensure generalizability.

Furthermore, a detailed status of migration (e.g., first- versus second-generation migration) was not assessed in the present study. Yet, this information could be used as a further predictor for cognitive performance, as second-generation immigrants may already be more integrated (e.g., reduction of language barriers). In the present study, we defined origin as “first language learned”. However, various definitions of migration background exist and they are often not comparable [46,47]. As a result, our study may not be comparable to other investigations. Further studies should include a more specific operationalization of migration and its different variants. For example, in the study conducted by Krist et al. (2017) [44], Turkish migrants were able to select the test language (German or Turkish version of the MoCA), and the group that picked the German version showed significantly better MoCA scores than those who were tested in the Turkish version. This result indicated that the level of acculturation and integration differed (with those picking German being more integrated) and that this level was associated with cognitive performance. Another systematic review on bilingualism as an influencing factor in test performance in older adults indicates that migration status, acculturation level, and language of neuropsychological testing need to be considered when assessing bilingual older adults [48]. In line with this, the systematic review on migration and cognition conducted by Xu et al. (2017) [9] demonstrated a significant association between a higher level of acculturation and better performance in cognitive functioning among migrants. However, a recent study showed that more data are needed to better evaluate the utility of measuring acculturation level in neuropsychological assessment [49].

One main strength of the present study is the inclusion of a German sample as a control group to compare predictors of cognitive performance between migrants and non-migrants. Another strength is that our study included both sociodemographic variables and depressive symptoms as possible influence factors on cognition.

## 5. Conclusions

The present study investigated sociodemographic and affective predictors of MoCA performance in German individuals and Turkish migrants living in Germany. The most important finding is that migration background and education have an impact on cognitive performance: individuals with a Turkish migration background and individuals with lower education scored lower on the MoCA. The need for culturally sensitive instruments that take the educational situation into account has also been noted in other studies. The influence of education on the MMST test performance was shown to be similarly high in a Turkish population in Denmark [50]. In addition, a recent study on diagnosing dementia in patients with a migration background among German general practitioners showed that a language barrier could significantly complicate the diagnostic process [51]. Therefore, culture-sensitive and education-adjusted screening tools for the assessment of cognitive functioning in this population group are of high importance and these factors should be considered when developing new tools for the assessment of cognitive functioning or normative data for the MoCA for specific subpopulations. Notably, some work has been done in this respect in recent years. For example, Goudsmit et al. (2017) [52] developed the Cross-Cultural Dementia Screening (CCD) as a culture-fair test for addressing these problems in a Turkish population living in the Netherlands. Furthermore, our own working group has developed a culture-sensitive dementia test battery tool for Turkish migrants living in Germany that is based on culture-fair nonverbal materials [53,54].

More generally, as predictors of MoCA scores in separate samples of German individuals and Turkish migrants differed according to age, education, sex, and depression with only education having predictive value in both populations, our study further emphasizes that normative studies for specific populations are important. For clinical practice, the results of the MoCA should be interpreted with caution. The results can provide initial information, but new and culture-specific tests are still needed. For example, further studies are needed that apply a new weighting of the MoCA subtests with demographically adjusted standard scores. A better alternative would be a validated culture-fair test battery that considers group-specific characteristics and cut-offs as well.

## Figures and Tables

**Table 1:** Characteristics of the German and Turkish samples.

	German Sample (*n* = 419)							Turkish Sample (*n* = 133)							*p*-Value
Sex	♂ = 169 (40.33%)				♀ = 250 (59.67%)			♂ = 67 (50.38%)				♀ = 66 (49.62%)			0.059
	Men		Women		*p*-Value	Total German Sample		Men		Women		*p*-Value	Total Turkish Sample		
	*M*	*SD*	*M*	*SD*		*M*	*SD*	*M*	*SD*	*M*	*SD*		*M*	*SD*	
Age	62.10	8.84	61.43	9.04	0.451	61.70	8.96	61.29	8.98	58.22	7.06	**0.030**	59.74	8.19	**0.020**
Education (in school years)	12.15	1.81	11.43	2.02	**<0.001**	11.72	1.97	10.47	4.09	6.92	4.91	**<0.001**	8.68	4.84	**<0.001**
Cognitive Status	27.08	2.23	27.43	2.47	0.134	27.29	2.38	25.79	3.12	24.43	4.63	**0.050**	25.11	3.40	**<0.001**
Depression	1.41	1.64	1.79	2.23	**0.048**	1.64	2.02	3.77	2.99	3.91	3.09	0.794	3.84	3.03	**<0.001**
Age classes (N%)															
≤54 years	22.5%	25.2%			24.1%			25.8%		41.8%		33.8%			
55–59 years	23.7%	26%			25.1%			28.8%		25.4%		27.1%			
60–64 years	19.5%	20%			19.8%			16.7%		11.9%		14.3%			
65–69 years	14.2%	8.4%			10.7%			12.1%		10.4%		11.3%			
70–74 years	10.7%	10.4%			10.5%			4.5%		7.5%		6%			
75–79 years	4.1%	5.6%			5%			9.1%		3%		6%			
80–84 years	4.1%	1.6%			2.6%			0%		0%		0%			
≥85 years	1.2%	2.8%			2.1%			3.0%		0%		1.5%			
Education															
Primary school *	0.6%	0.4%			0.5%			21.2%		53.7%		37.6%			
Secondary Education	7.1%	15.2%			11.9%			39.4%		22.4%		30.8%			
Higher Education	91.7%	84%			87.1%			39.4%		23.9%		31.6%			

Note. Cognitive status was measured using the Montreal Cognitive Assessment Test (MoCA; Nasreddine et al., 2005 [13]) with a maximum of 30 points (German sample: 31 points, including correction for education). Depression was measured using the Geriatric Depression Scale (GDS; Yesavage et al., 1983 [26]) with a maximum of 15 points. * Primary school in Germany = 0–4 years; primary school in Turkey = 0–5 years.

**Table 2:** Linear regression analysis for German sample on cognition measured with MoCA (*n* = 417).

	*B*	*SE B*	*ß*	*t*	*p*
Constant	26.52	1.27		20.87	<0.001
Education	0.36	0.06	0.29	5.86	<0.001
Age	−0.05	0.01	−0.19	−3.95	<0.001
Sex	−0.46	0.23	−0.09	−2.06	0.040
Depression	−0.21	0.05	−0.17	−3.91	<0.001
R^2^ = 0.200; adjusted R^2^ = 0.192; *p* < 0.001					

**Table 3:** Linear regression analysis for Turkish sample on cognition measured with MoCA (*n* = 133).

	*B*	*SE B*	*ß*	*t*	*p*
Constant	20.44	2.94		6.94	<0.001
Education	0.33	0.08	0.40	4.07	<0.001
Age	0.01	0.04	0.03	0.30	0.765
Sex	0.36	0.74	0.05	0.48	0.630
Depression	0.07	0.12	0.05	0.65	0.519
R^2^ = 0.163; corrected R^2^ = 0.137; *p* < 0.001					

**Table 4:** Linear regression analysis for German and Turkish sample on Cognition measured with MoCA (*n* = 264).

	*B*	*SE B*	*ß*	*t*	*p*
Constant	22.04	1.68		13.14	<0.001
Ethnicity	1.14	0.46	0.17	2.49	0.013
Education	0.34	0.06	0.40	5.92	<0.001
Age	−0.01	0.02	−0.01	−0.22	0.827
Sex	−0.61	0.40	−0.09	−1.54	0.125
Depression	0.03	0.08	0.03	0.45	0.655
R^2^ = 0.226; adjusted R^2^ = 0.211; *p* < 0.001					

## Data Availability

Data sharing not applicable.
